# Characterization of Patient Activation among Childhood Cancer Survivors in the St. Jude Lifetime Cohort Study (SJLIFE)

**DOI:** 10.3390/cancers16183220

**Published:** 2024-09-21

**Authors:** Megan E. Ware, Angelica De La Cruz, Qian Dong, Kyla Shelton, Tara M. Brinkman, I-Chan Huang, Rachel Webster, Brian Potter, Kevin Krull, Sedigheh Mirzaei, Matthew Ehrhardt, Melissa M. Hudson, Gregory Armstrong, Kirsten Ness

**Affiliations:** 1 Department of Epidemiology and Cancer Control, St. Jude Children’s Research Hospital, Memphis, TN 38105, USAmelissa.hudson@stjude.org (M.M.H.);; 2 Department of Kinesiology, Health Promotion, and Recreation, University of North Texas, Denton, TX 76201, USA; 3 Department of Biology, University of Puerto Rico—Rio Piedras Campus, San Juan, PR 00925, USA; 4 Department of Psychology and Biobehavioral Sciences, St. Jude Children’s Research Hospital, Memphis, TN 38105, USA; 5 Department of Biostatistics, St. Jude Children’s Research Hospital, Memphis, TN 38105, USA; 6 Department of Oncology, St. Jude Children’s Research Hospital, Memphis, TN 38105, USA

**Keywords:** patient activation, childhood cancer survivors, health behaviors, psychological health

## Abstract

**Simple Summary:**

Patient activation is a very important psychological construct to examine in individuals who have chronic conditions, because it assesses the one’s confidence in managing their own health and care. For childhood cancer survivors, continued follow-up is imperative to monitor late effects conditions; yet many do not adhere to surveillance guidelines. Therefore, investigating this construct could highlight risk factors in the population that contribute to low activation. Furthermore, examining the long-term impact of patient activation on psychological health as well as its contribution to health behavior could provide a reasonable target for interventions to enhance health outcomes in survivors.

**Abstract:**

Background: Patient activation describes a willingness to take action to manage health and is associated with health outcomes. The purpose of this study was to characterize patient activation and its association with psychological outcomes and health behaviors in childhood cancer survivors. Methods: Participants were from the St. Jude Lifetime Cohort Study (SJLIFE). Activation levels (1–4, 4 = highest activation) were measured with the Patient Activation Measure (PAM). Psychological outcomes and health behaviors were obtained via self-report. Cognitive function was assessed by trained examiners. ANOVA or chi-squared tests were utilized to assess group-level differences in activation. Multivariable regression models were used to assess associations between PAM scores and outcomes of interest. Results: Among 2708 survivors and 303 controls, more survivors endorsed lower activation levels than the controls (11.3 vs. 4.7% in level 1) and fewer survivors endorsed the highest level of activation than the controls (45.3 vs. 61.5% in level 4). Not endorsing depression (OR: 2.37, 95% CI 1.87–2.99), anxiety (OR: 2.21, 95% CI 1.73–2.83), and somatization symptoms (OR: 1.99, 95% CI 1.59–2.50), general fear (OR: 1.45, 95% CI 1.23–1.71) and body-focused (OR: 2.21, 95% CI 1.83–2.66), cancer-related worry, and physical (OR: 2.57, 95% CI 2.06–3.20) and mental (OR: 2.08, 95% CI 1.72–2.52) HRQOL was associated with higher levels of activation. Lower activation was associated with not meeting physical activity guidelines (OR: 2.07, 95% CI 1.53–2.80). Conclusions: Survivors endorsed lower activation levels than peers. Interventions to improve physical and psychological health outcomes could leverage these results to identify survivors who benefit from support in patient activation.

## 1. Introduction

Childhood cancer survival rates have increased in recent decades due to a better understanding of cancer biology, improvements in diagnostic technology, and the development of effective, risk-stratified treatment strategies [1,2]. However, treatment has lasting impacts, and survivors face continued challenges to their health as late effects (health complications that develop after cancer treatment has ended) develop and progress throughout survivorship; some of these late effects include conditions such as congestive heart failure, coronary artery disease, stroke, renal failure, and second malignant neoplasms [3,4]. These late effects have serious implications for survivors’ long-term health and mortality [5,6] as well as psychological well-being and quality of life [7,8]. Data suggest that engaging in optimal health behaviors, such as engaging in physical activity and refraining from smoking, risky drinking, and illicit drug use, decreases the risk of adverse health outcomes [9,10] and improves psychological well-being and quality of life [11]. Survivors do not always engage in healthy behaviors, even when provided with an adequate education to understand the future risk of poor engagement [12,13,14]. The reasons for a lack of engagement are likely multiple. However, one reason may be that they lack the skills and confidence to manage their own health and healthcare. If this is the case, care models need to be developed to provide survivors with these skills. 

Patient activation is defined as the “skills and confidence a person has in managing their own health and health care” [15], which focuses on a patient’s “willingness and ability to take independent actions to manage their health and care” [16]. This is incredibly important, as patient involvement has been linked to improved health outcomes and improved outcomes of healthcare in patients who are more involved in their care [15,16,17,18,19]. However, survivors face challenges as they transition from pediatric or adolescent care to adult care; a retrospective study of 370 survivors (median age at diagnosis 10.2 years (range 1–21 years) found the probability of continued engagement in long-term follow-up 6 to 10 years from treatment completion to be 68.5%, dropping to 47.7% by years 11 to 15 [20]. One potential framework with which to examine this decline is outlined in the Life Course Health Development (LCHD) framework, in which it is posited that biopsychosocial influences affect an individual’s health trajectories in different manners across the lifespan [21]. In line with the LCHD, a survivor’s cancer-specific or individual challenges could be further exacerbated by socioeconomic status, health behaviors, and familial or other close relationships in different ways across the lifespan [22]. In combination, these factors could contribute to lower levels of activation, but could also be exacerbated by lower levels of activation, leading to poor health outcomes in survivors.

Associations between patient activation, health behaviors, and psychological outcomes have not been explored in a large cohort of childhood cancer survivors. Understanding the risk factors among survivors for low activation, and the potential contribution of patient activation to health behaviors and psychological outcomes will provide insight for the design of interventions to promote optimal health behaviors among survivors. Therefore, the aim of this study is to characterize patient activation in the St. Jude Lifetime Cohort Study (SJLIFE) and to identify the associations between, as follows: (1) patient activation and health behaviors; and (2) psychological factors and patient activation to identify factors associated with low activation. 

## 2. Materials and Methods

### 2.1. Participants

Participants in this study were enrolled in the St. Jude Lifetime Cohort Study (SJLIFE) [23,24], a study that includes childhood cancer survivors treated at St. Jude Children’s Research Hospital between 1962 and 2012, who were at least 5 years from their primary cancer diagnosis, and at least 18 years of age at assessment. A comparison group comprised of a community who did not have childhood cancer was also included to assess differences in patient activation level between otherwise healthy adults and survivors. Eligible participants for this analysis were members of the cohort who completed survey assessments at a single cross-sectional timepoint. Study measures and documents were approved by the SJCRH Institutional Review Board.Participants provided written informed consent prior to study activities.

### 2.2. Measures

#### 2.2.1. Patient Activation

Patient activation was measured using the short-form Patient Activation Measure (PAM) [18]. The short form of the PAM is a 13-item measure instructing participants to report personal levels of agreement or disagreement with statements related to knowledge, skill, and confidence for self-managing their own health and healthcare. Responses to items are Likert-scored on a 0 to 4 scale, with 0 indicating “disagree strongly”, 1 indicating “disagree”, 2 indicating “agree”, 3 indicating “agree strongly”, and 4 indicating “not applicable” [18]. PAM short-form raw scores are calculated as follows: total score = [raw score]/[number of items answered excepting “non applicable” items] × 13. Raw scores are converted to four activation levels [25]: Level 1 (Score 0.0–47.0)—“People are passive and feel overwhelmed about managing their health. They may be unprepared to take an active role”; Level 2 (Score 47.1–55.1)—“People may lack specific knowledge and confidence to self-manage their health”; Level 3—(Score 55.2–67.0) “People are beginning to take action but may lack the confidence and skill to sustain the activity”; Level 4 (Score 67.1–100.0)—“People have adopted behaviors to support their health, but may not be able to maintain them over time when they are facing life stressors” [26].

#### 2.2.2. Psychological Factors

Anxiety, somatization, and depression symptoms were assessed using the Brief Symptom Inventory (BSI-18) [27]. T-scores were created for each participant, with scores ≥ 63 (top 10th percentile) classified as elevated anxiety, somatization, and depression symptoms [28]. Physical and mental-health-related quality of life (HRQOL) were assessed using the 8 subscales measuring general health, physical function, role limitations caused by physical factors, bodily pain, social function, mental health, role limitations caused by emotional factors, and vitality from the Short-Form Health Survey (SF-36) [29]. T-scores were created for each participant, with scores ≤ 40 representing poor HRQOL. 

Cancer-related worry (CRW) was assessed via self-report using six questionnaire items: (1) “I have general fears about cancer”, (2) “I am worried about my cancer coming back”, (3) “I mostly worry about my cancer and its treatment right before I go for a check-up”, (4) “I am concerned about physical problems related to my cancer”, (5) “I am worried about my appearance”, and (6) “Do you currently have anxieties/fears as a result of your cancer or similar illness, or it’s treatment?”. Factor analysis was employed to create two independent CRW factors, as follows: body-focused and general fear. The averages of respective items were calculated to create factor scores, which were categorized as <3 (not endorsing CRW) and ≥3 (endorsing CRW) [30].

#### 2.2.3. Health Behavior

Physical activity (PA) was assessed by self-report of the frequency and amount of moderate to vigorous intensity PA per week. Self-report values were then categorized into meeting or not meeting Centers for Disease Control and Prevention (CDC) PA guidelines of 150 min of moderate or 75 min of vigorous PA per week [31]. Smoking status was captured via self-report, with those endorsing current smoking (within the past 30 days) being categorized as smokers. Heavy or risky drinking was captured via self-report and categorized into endorsing or not endorsing the behavior according to the National Institute on Alcohol Abuse and Alcoholism (NIAAA) criteria for risky drinking (>3 per day or >7 per week (females) >4 per day or >14 per week (males)). Diet quality was assessed via the Healthy Eating Index (HEI) [25]. HEI scores were categorized into <51 (poor diet), 50–80 (needs improvement), and >80 (good). Sleep quality was assessed using the PROMIS Sleep Disturbance, 8a [27]. T-scores were calculated for the sample, and then categorized into <25 (none to slight), 25> and ≤30 (mild), and >30 (moderate to severe).

#### 2.2.4. Sociodemographic and Clinical Covariates

Personal sociodemographic data were self-reported and included sex, gender, self-identified race/ethnicity, age at assessment, insurance status (uninsured vs. insured), and educational attainment (high school or less, some post-high school, college degree or higher). Neighborhood-level socioeconomic status was assessed using the Area Deprivation Index (ADI), which is a composite measure derived from American Community Survey components reflective of 17 neighborhood-level SES measures within US Census blocks [32]. Each block was assigned a percentile and quartiles were created, with lower quartiles representing higher socioeconomic disadvantage. Clinical data were abstracted from medical records and included age at diagnosis and primary diagnosis. Chronic condition presence and severity was assessed for each participant utilizing the Common Terminology Criteria for Adverse Events (CTCAE) [33] grading of 13 organ systems. Those with grades of 3 or more in any of the systems were considered as having a condition within the respective system. Perceived instrumental support was assessed using the PROMIS Instrumental Support, 6a [34]. T-scores were calculated for each participant. 

#### 2.2.5. Cognitive Function Assessments 

Testing of intelligence [35], executive functioning [36], attention [37], processing speed [36,38], and memory [39] were completed in standardized order and administered by certified examiners under the supervision of a board-certified clinical neuropsychologist. Scores were referenced to national normative sample data to generate age-adjusted Z scores, with mild impairment representing more than −1.5 to −1.0, moderate impairment representing more than −2.0 to −1.5, or severe impairment representing −2.0 or less [40]. Only intelligence was selected as an eligible covariate for analysis.

### 2.3. Analyses

Descriptive statistics were calculated to characterize the sample. Distributions of activation level across both survivors and controls were compared using the chi-squared test. Further analyses focused on the distribution of activation level across demographic and clinical factors in survivors only and were performed using chi-squared or AVOVA tests. Multivariable logistic regression models, adjusted for demographic and clinical covariates, were used to assess the associations between patient activation and health behaviors in survivors only; multivariable ordinal logistic regression models were used to assess the associations between psychological factors and patient activation in survivors only. Covariates with *p* < 10 in univariate analyses were selected for the multivariable models. Patient activation levels, age at patient activation assessment, age at diagnosis, sex, primary cancer diagnosis, and educational attainment were retained in all models. Categorical variables indicating patient activation level were utilized in analyses. All analyses were completed in SAS (SAS 9.4, Cary, NC, USA).

## 3. Results

**Figure 1:**
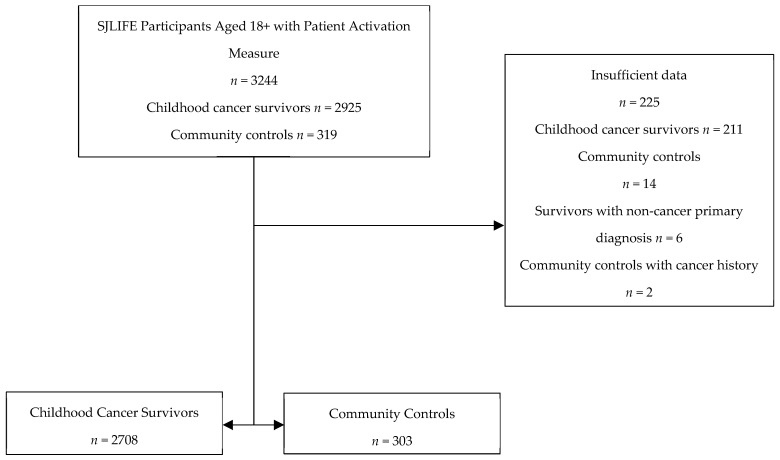
Consort diagram.

A total of 2708 survivors and 303 controls were eligible for analysis. Those with insufficient data (did not provide responses for survey assessments), survivors with a non-cancer primary diagnosis, and controls with a prior cancer history, were excluded from analyses (Figure 1). Survivors differed from controls on distributions of sex (50.6 vs. 41.6% male), race/ethnicity (82.0 vs. 80.2% non-Hispanic White), educational attainment (40.9 vs. 58.2% college degree or higher), and ADI quartile (23.3 vs. 36.3% quartile 1). Survivors also differed from the controls with respect to the presence of cardiovascular (8.6 vs. 1.7%), endocrine (37.5 vs. 24.8%), auditory (12.8 vs. 2.0%), ocular (10.6 vs. 2.0%), neurological (5.8 vs. 3.0%), and sexual or reproductive conditions (10.0 vs. 1.0%). Survivors were, on average, older than the controls at survey (33.8 ±10.5 vs. 30.7 ± 9.8 years). The largest percentage of survivors was diagnosed with leukemia as a primary cancer (32.0%), followed by lymphoma (18.2%), CNS tumor (15.6%), sarcoma (13.2%), embryonal (12.4%), and other cancers (8.5%). Over half of the survivors received radiation (53.6%), chemotherapy (83.7%), and surgery (93.5%) during treatment (Table 1). 

Survivors differed from the controls in distributions of patient activation, with a larger percentage of survivors endorsing lower levels of patient activation than the controls (11.3 vs. 4.7% in level 1 and 13.8 vs. 9.7% in level 2) and a larger percentage of the controls endorsing the highest level of patient activation than survivors (61.5 vs. 45.3%) (Table 1).Among survivors only, differences in patient activation were observed by diagnosis, where a larger percentage of those with a history of lymphoma or CNS tumors endorsed level 1 rather than level 4 activation (19.4 vs. 18.9% and 27.3 vs. 13.2%). A larger percentage of those treated with radiation reported level 1 rather than level 4 activation, compared to those who were not treated with radiation (60.2% vs. 50.8%). A larger percentage of survivors with cardiovascular, endocrine, immune, neurological, and auditory conditions endorsed level 1 patient activation rather than level 4 activation. Distributions of educational attainment, insurance coverage, and ADI quartile differed between patient activation levels, such that persons with a high school or lower attainment, who were uninsured, or who lived in ADI quartile 4 were more likely to endorse level 1 vs. level 4 patient activation (Table 2). Across cognitive function covariates, distributions of impairment differed between patient activation levels, such that a higher percentage of persons in the impaired function categories endorsed level 1 vs. level 4 patient activation (Table 3).

Survivors in levels 1 and 4 patient activation differed in distributions of health behaviors, such that those who endorsed currently smoking (18% vs. 12%), did not meet CDC PA guidelines (56% vs. 32%), and those who had poor diet quality (31% vs. 18%) were more likely to endorse level 1 vs. 4 patient activation (Table 4). The opposite trend was observed with respect to risky drinking, wherein a larger percentage of those who endorsed level 4 (vs. level 1) activation also reported risky drinking (37% vs. 23%). Moderate or severe sleep disturbance did not differ across patient activation levels (9.3% in level 1 vs. 9.8% in level 4, *p* = 0.42). Across all psychological factors, distributions of impairment differed by patient activation levels. The percentages of those who reported depression symptoms (28% vs. 7%), anxiety symptoms (24% vs. 7%), somatization symptoms (29% vs. 9%), general (42% vs. 29%) and body-focused (42% vs. 16%) CRW, and suboptimal physical (40% vs. 10%) and mental (40% vs. 15%) health-related quality of life were of a larger proportion for level 1 patient activation than for level 4 (Table 5).

Patient activation levels were not associated with risky drinking, smoking, diet quality, or sleep disturbance (Supplementary Tables S1–S4). However, in multivariable models adjusted for age at survey, age at diagnosis, race, educational attainment, gender, diagnosis, intelligence, and CTCAE grade 3+ cardiovascular, endocrine, auditory, hematological, neurological, pulmonary, and renal conditions, the endorsing of patient activation levels 3 vs. 1 (OR: 1.53, 95% CI: 1.13–2.09) or 4 vs. 1 (OR: 2.07, 95% CI: 1.53–2.80) was associated with meeting CDC PA guidelines (Table 6).

Multivariable ordinal logistic regression models assessing associations between psychological factors (respectively) and patient activation were adjusted for age at survey, age at diagnosis, race, educational attainment, gender, diagnosis, insurance status, perceived instrumental support, and CTCAE grade 3+ auditory, cardiovascular, endocrine, immunologic, neurological, and pulmonary conditions. Compared to those who endorsed symptoms of anxiety, depression, somatization, general fear CRW, and body-focused CRW, those who did not endorse symptoms of anxiety (OR: 2.21, 95% CI 1.73–2.83), depression (OR: 2.37, 95% CI 1.87–2.99), somatization (OR: 1.99, 95% CI 1.59–2.50), general fear (OR: 1.45, 95% CI 1.23–1.71) and body-focused (OR: 2.21, 95% CI 1.83–2.66) CRW, and physical (OR: 2.57, 95% CI 2.06–3.20) and mental (OR: 2.08, 95% CI 1.72–2.52) HRQOL had greater odds of endorsing higher levels of patient activation (Table 7).

## 4. Discussion

This study characterized patient activation, a construct related to more positive health outcomes, in a large sample of childhood cancer survivors. The study findings highlight demographic and clinical factors placing survivors at higher risk for low patient activation, such as a primary diagnosis of lymphoma and CNS tumor, receiving radiation therapy as part of treatment, and the presence of cardiovascular, endocrine, immune, neurological, and auditory chronic conditions. Additionally, social factors, such as lower educational attainment, a lack of insurance, and lower socioeconomic status, were identified as risk factors for low patient activation in this population. These data are essential for identifying survivors who may benefit from interventions to improve health outcomes, which is of concern because of this population’s vulnerability to adverse health outcomes. Using an LCHD lens, these results lend themselves to identifying how patient activation contributes to survivors’ health trajectories in a biopsychosocial context.

The extant literature demonstrates evidence of differing survival outcomes among diagnosis groups, which could be an underpinning of lower patient activation among certain diagnosis groups in the present study. Numerous studies have highlighted long-term functional, neurocognitive, and social outcomes of childhood CNS tumor survival, indicating that survivors of CNS tumors are at risk of not achieving independence as adults, neurocognitive impairment, and delayed or disrupted attainment of adult social milestones [41,42]. Additionally, there is evidence to indicate that survivors of lymphoma experience a similar risk of impaired neurocognitive function, which is associated with lower attainment of social milestones [43]. Neurocognitive impairment in both of these groups could lead to lower educational attainment and fewer downstream opportunities for socioeconomic attainment (i.e., employment, independent living, obtaining insurance coverage), all of which synergistically contribute to patient activation [44]. Unfortunately, because of the cross-sectional nature of the present study, no inference can be made with regard to the directionality of patient activation and psychological functioning; future studies should seek to address the causal nature of these associations. However, the present results could be utilized to identify survivors with psychological comorbidities who need more resources to manage self-health and care. 

A novel finding in the present study is the association between ADI and patient activation, such that those in less desirable ADIs also reported lower patient activation. The direction of this association is as expected, sheds light on the potential role of SES or SES-related factors in the development of a survivors’ patient activation and is in line with some findings in the extant literature. In a study of prospective spine surgery patients, increased patient activation was associated with an annual income >$80,000 compared to <$30,000 (OR 1.72, 95% CI 1.18–2.50, *p* = 0.01), and reporting employment compared to not reporting employment (OR 1.37, 95% CI 1.03–1.84, *p* = 0.03) [45]. Similarly, a study conducted in the United States on a random sample of chronically ill adults found that employment (β = 3.11, *p* < 0.001) and an income >$75,000 (β = 2.22, *p* < 0.001) were associated with higher levels of patient activation compared to unemployment and an income of <$35,000 [46]. It is important to note, however, that in a sample of older adults, socioeconomic characteristics only explained 5–6% of the variations in patient activation scores [47]; therefore, further research should continue to examine other factors that could influence variations in patient activation measures beyond socioeconomic characteristics.

The investigation of health behaviors across patient activation levels was a novel aspect of the present study. While the frequencies of engagement in suboptimal health behaviors differed across patient activation levels in expected patterns, after adjustment for relevant covariates, associations were only seen between patient activation levels and PA. Similarly, in a study of patient activation in older adults with multimorbidity, the authors found positive associations between patient activation levels and PA (posterior probability: 0.847, Bayes factor: 5.54), and between patient activation level and medication adherence (posterior probability: 0.059, Bayes factor: 0.063) [48], but not between patient activation levels and either smoking or diet quality [48]. Similar observations were made in a sample of persons with atrial fibrillation; patients endorsing the highest level of patient activation also endorsed more physical activity than those endorsing the second highest level [49].

The associations between patient activation and physical activity and patient activation and psychological factors in multivariate analyses indicate that increased patient activation is linked to more optimal behavioral and psychological factors, even with adjustment for factors like SES. Within the context of LCHD, patient activation could be a potential protective psychosocial factor on a survivor’s health trajectory [22]. Therefore, maximizing patient activation could lead to better health outcomes in survivors. Commonly utilized strategies to address patient activation during interventions to improve health-related outcomes include problem solving, feedback, individualized care plans, peer support, health advisement, theory-based counseling, and skill-building [50]. One, or several of these types, of these strategies have been utilized in interventions delivered remotely [51,52,53,54,55], face-to-face [56,57,58], and in hybrid [59,60,61,62] models in various populations that experience chronic disease and/or disability, including childhood cancer survivors [63,64]. Studies of patient activation interventions on self-managed health behaviors in patients with type II diabetes have demonstrated increases in PA post-patient activation interventions [65,66,67,68]. These studies utilized several models of interventions on patient activation, including diabetes education and patient empowerment [65], motivational interviewing [66], education on medication adherence and lifestyle changes [67], and goal-setting and monitoring [68].

This study is not without limitations. The limited ethnic diversity in our study cohort underscores the need for evaluations of patient activation in more diverse samples. Health behaviors were obtained via self-report; while this type of ascertainment is typical, responses can be subject to social desirability bias. Associations between patient activation and anxiety, depression, and somatization symptoms must be interpreted with caution, as the BSI-18 is a symptom inventory and does not diagnose anxiety, depression, and somatization. Longitudinal healthcare utilization, follow-up care engagement, and adverse clinical outcomes were not examined in this study; future studies are warranted to evaluate associations between patient activation and these long-term outcomes in survivors.

## 5. Conclusions

Survivors enrolled in SJLIFE endorsed lower patient activation levels than the controls. These results highlight unique risk factors for low patient activation and the psychological contributors to low patient activation and highlight the role of patient activation as a contributor to health behavior. Further interventions to improve engagement in optimal health behaviors and improve psychological health could leverage these results to identify survivors who would benefit from support in patient activation.

## Figures and Tables

**Table 1:** Demographic characteristics of survivors and controls.

Characteristic	Survivors (*n* = 2708)	Controls (*n* = 303)	*p* Value
	No. (%)	No (%)	
Sex			0.0003
Male	1370 (50.6)	126 (41.6)	
Female	1338 (49.4)	177 (58.4)	
Race and Ethnicity			<0.0001
Non-Hispanic White	2220 (82.0)	243 (80.2)	
Non-Hispanic Black	380 (14.0)	20 (6.6)	
Others	108 (4.0)	40 (13.2)	
Primary Cancer Diagnosis			
Leukemia	867 (32.0)	--	
Lymphoma	464 (18.2)	--	
Sarcoma	358 (13.2)	--	
CNS Tumor	423 (15.6)	--	
Embryonal	337 (12.4)	--	
Others	229 (8.5)	--	
Radiation			
Yes	1452 (53.6)	--	
No	1256 (46.4)	--	
Chemotherapy			
Yes	2267 (83.7)	--	
No	441 (16.3)	--	
Surgery			
Yes	2531 (83.7)	--	
No	177 (6.5)	--	
CTCAE ^1^ Grade 3+ Conditions			
Cardiovascular			<0.0001
Yes	232 (8.6)	5 (1.7)	
No	2476 (91.4)	298 (98.3)	
Endocrine			<0.0001
Yes	1015 (37.5)	75 (24.8)	
No	1693 (62.5)	228 (75.2)	
Pulmonary			0.32
Yes	203 (7.5)	18 (5.9)	
No	2505 (92.5)	285 (94.1)	
Musculoskeletal			0.50
Yes	23 (0.8)	1 (0.3)	
No	2685 (99.2)	302 (99.7)	
Neurological			0.04
Yes	158 (5.8)	9 (3.0)	
No	2550 (94.2)	294 (97.0)	
Auditory			<0.0001
Yes	346 (12.8)	6 (2.0)	
No	2362 (87.2)	297 (98.0)	
Gastrointestinal			0.17
Yes	38 (1.4)	1 (0.3)	
No	2670 (98.6)	302 (99.7)	
Hematologic			1.00
Yes	8 (0.3)	0 (0.0)	
No	2700 (99.7)	303 (100.00)	
Immunologic			1.00
Yes	24 (0.9)	2 (0.7)	
No	2684 (99.1)	301 (99.3)	
Ocular			<0.0001
Yes	286 (10.6)	6 (2.0)	
No	2442 (89.4)	297 (98.0)	
Renal			0.07
Yes	31 (1.1)	0 (0.0)	
No	2677 (98.9)	303 (100.0)	
Reproductive			<0.0001
Yes	270 (10.0)	3 (1.0)	
No	2438 (99.0)	300 (99.0)	
Subsequent neoplasm			0.16
Yes	24 (0.9)	0.0 (0.0)	
No	2684 (99.1)	303 (100.0)	
Educational Attainment			<0.0001
High school or less	665 (26.0)	38 (12.8)	
Some post-high school	847 (33.1)	86 (29.1)	
College degree or higher	1047 (40.9)	172 (58.1)	
Insurance Coverage			0.43
Insured	2379 (88.5)	272 (90.1)	
Uninsured	308 (11.5)	30 (9.9)	
ADI ^2^ Quartiles			<0.0001
Quartile 1	631 (23.3)	110 (36.3)	
Quartile 2	679 (25.1)	84 (27.7)	
Quartile 3	707 (26.1)	64 (21.1)	
Quartile 4	691 (25.5)	45 (14.9)	
Patient Activation Level			<0.0001
Level 1 ^3^	304 (11.3)	14 (4.7)	
Level 2 ^4^	371 (13.8)	29 (9.7)	
Level 3 ^5^	800 (29.7)	72 (24.1)	
Level 4 ^6^	1220 (45.3)	184 (61.5)	
	Mean (SD)	Mean (SD)	
Age at diagnosis (y)	8.9 (5.8)	--	--
Age at evaluation	33.8 (10.5)	30.7 (9.8)	<0.0001

^1^ CTCAE: Common Terminology Criteria for Adverse Events. ^2^ ADI: Area Deprivation Index. ^3^ People are passive and feel overwhelmed about managing their health. They may be unprepared to take an active role. ^4^ People may lack specific knowledge and confidence to self-manage their health. ^5^ People are beginning to take actions but may lack the confidence and skill to sustain the activity. ^6^ People have adopted behaviors to support their health but may not be able to maintain them over time when they are facing life stressors.

**Table 2:** Distribution of survivor demographic characteristics across patient activation levels.

Characteristic	Level 1 (*n* = 304)	Level 2 (*n* = 371)	Level 3 (*n* = 800)	Level 4 (*n* = 1220)	*p* Value
	No. (%)	No (%)	No (%)	No. (%)	
Sex					0.33
Male	150 (49.3)	194 (52.3)	421 (52.6)	596 (48.9)	
Female	154 (50.7)	177 (47.7)	379 (47.4)	624 (51.1)	
Race and Ethnicity					
Non-Hispanic White	250 (82.2)	302 (81.4)	664 (83.0)	995 (81.6)	
Non-Hispanic Black	44 (14.5)	52 (14.0)	104 (13.0)	176 (14.4)	
Others	10 (3.3)	17 (4.6)	32 (4.0)	49 (4.0)	0.95
Primary Cancer Diagnosis					<0.0001
Leukemia	80 (26.3)	130 (35.0)	260 (32.5)	393 (32.2)	
Lymphoma	59 (19.4)	62 (16.7)	140 (17.5)	230 (18.9)	
Sarcoma	34 (11.2)	57 (15.4)	107 (13.4)	159 (13.0)	
CNS Tumor	83 (27.3)	55 (14.8)	121 (15.1)	161 (13.2)	
Embryonal	34 (11.2)	35 (9.4)	105 (13.1)	163 (13.4)	
Others	14 (4.6)	32 (8.6)	67 (8.4)	114 (9.3)	
Radiation					0.01
Yes	183 (60.2)	211 (56.9)	430 (53.8)	620 (50.8)	
No	121 (39.8)	160 (43.1)	370 (46.3)	600 (49.2)	
Chemotherapy					0.11
Yes	247 (81.3)	323 (87.1)	658 (82.3)	1029 (84.3)	
No	57 (18.8)	48 (12.9)	142 (17.8)	191 (15.7)	
Surgery					0.23
Yes	287 (94.4)	354 (95.4)	739 (92.4)	1139 (93.4)	
No	17 (5.6)	17 (4.6)	61 (7.6)	81 (6.6)	
CTCAE ^1^ Grade 3+ Conditions					
Cardiovascular					0.004
Yes	24 (7.9)	44 (11.9)	81 (10.1)	82 (6.7)	
No	280 (92.1)	327 (88.1)	719 (89.9)	1138 (93.3)	
Endocrine					0.005
Yes	130 (42.8)	161 (43.4)	290 (36.3)	427 (35.0)	
No	174 (57.2)	210 (56.6)	510 (63.8)	793 (65.0)	
Pulmonary					0.07
Yes	33 (10.9)	29 (7.8)	60 (7.5)	78 (6.4)	
No	271 (89.1)	342 (92.2)	740 (92.5)	1142 (93.6)	
Musculoskeletal					0.90
Yes	3 (1.0)	3 (0.8)	8 (1.0)	9 (0.7)	
No	301 (99.0)	368 (99.2)	792 (99.0)	1211 (99.3)	
Neurological					<0.0001
Yes	42 (13.8)	17 (4.6)	49 (6.1)	47 (3.9)	
No	262 (86.2)	354 (95.4)	751 (93.9)	1173 (96.1)	
Auditory					<0.0001
Yes	67 (22.0)	49 (13.2)	103 (12.9)	126 (10.3)	
No	237 (78.0)	322 (86.8)	697 (87.1)	1094 (89.7)	
Gastrointestinal					0.82
Yes	5 (1.6)	6 (1.6)	9 (1.1)	18 (1.5)	
No	299 (98.4)	365 (98.4)	791 (98.9)	1202 (98.5)	
Hematologic					0.48
Yes	1 (0.3)	1 (0.3)	4 (0.5)	2 (0.2)	
No	303 (99.7)	370 (99.7)	796 (99.5)	1218 (99.8)	
Immunologic					0.01
Yes	3 (1.0)	9 (2.4)	6 (0.8)	6 (0.5)	
No	301 (99.0)	362 (97.6)	794 (99.3)	1214 (99.5)	
Ocular					0.28
Yes	32 (10.5)	41 (11.1)	97 (12.1)	115 (9.4)	
No	272 (89.5)	330 (88.9)	703 (87.9)	1105 (90.6)	
Renal					0.70
Yes	4 (1.3)	6 (1.6)	9 (1.1)	12 (1.0)	
No	300 (98.7)	365 (98.4)	791 (98.9)	1208 (99.0)	
Reproductive					0.83
Yes	34 (11.2)	35 (9.4)	83 (10.4)	118 (9.7)	
No	270 (88.8)	336 (90.6)	717 (89.6)	1102 (90.3)	
Subsequent neoplasm					0.84
Yes	3 (1.0)	3 (0.8)	5 (0.6)	12 (1.0)	
No	301 (99.0)	368 (99.2)	795 (99.4)	1208 (99.0)	
Educational Attainment					<0.0001
High school or less	116 (41.9)	100 (28.3)	223 (29.3)	221 (19.1)	
Some post-high school	94 (33.9)	129 (36.5)	273 (35.8)	348 (30.0)	
College degree or higher	67 (24.2)	124 (35.1)	266 (34.9)	590 (50.9)	
Insurance Coverage					0.01
Insured	261 (87.0)	321 (87.2)	688 (86.3)	1098 (90.8)	
Uninsured	39 (13.0)	47 (12.8)	109 (13.7)	111 (9.2)	
ADI ^2^ Quartiles					<0.0001
Quartile 1	51 (16.8)	73 (19.7)	171 (21.4)	335 (27.5)	
Quartile 2	77 (25.3)	95 (25.6)	187 (23.4)	318 (26.1)	
Quartile 3	81 (26.6)	94 (25.3)	235 (29.4)	293 (24.0)	
Quartile 4	95 (31.3)	109 (29.4)	207 (25.9)	274 (22.5)	
	Mean (SD)	Mean (SD)	Mean (SD)	Mean (SD)	
Age at diagnosis (y)	8.7 (5.6)	9.5 (5.9)	8.8 (5.7)	8.9 (5.7)	0.17
Age at evaluation	33.6 (10.3)	35.3 (11.3)	33.7 (10.8)	33.5 (10.1)	0.04

^1^ CTCAE: Common Terminology Criteria for Adverse Events. ^2^ ADI: Area Deprivation Index.

**Table 3:** Distribution of survivor cognitive function across patient activation levels.

Domain	Level 1	Level 2	Level 3	Level 4	*p* Value
	No. (%)	No (%)	No (%)	No. (%)	
Intelligence					<0.0001
No impairment	180 (65.5)	282 (84.4)	621 (83.9)	996 (89.3)	
Mild impairment	33 (12.0)	24 (7.2)	60 (8.1)	65 (5.8)	
Moderate impairment	16 (5.8)	13 (3.9)	29 (3.9)	30 (2.7)	
Severe impairment	46 (16.7)	15 (4.5)	30 (4.1)	24 (2.2)	
Attention					
Focused Attention					<0.0001
No impairment	198 (66.7)	308 (84.6)	685 (87.5)	1078 (89.6)	
Mild impairment	18 (6.06)	14 (3.85)	30 (3.8)	49 (4.07)	
Moderate impairment	14 (4.7)	8 (2.2)	19 (2.4)	20 (1.7)	
Severe impairment	67 (22.6)	34 (9.3)	49 (6.3)	56 (4.7)	
Sustained Attention					<0.0001
No impairment	199 (71.1)	308 (85.8)	643 (83.8)	1040 (88.2)	
Mild impairment	19 (6.8)	18 (5.0)	47 (6.1)	56 (4.8)	
Moderate impairment	18 (6.4)	12 (3.4)	21 (2.7)	26 (2.2)	
Severe impairment	44 (15.7)	21 (5.9)	56 (7.3)	57 (4.8)	
Attention Span					<0.0001
No impairment	198 (65.8)	288 (78.7)	642 (81.1)	1005 (83.2)	
Mild impairment	52 (17.3)	54 (14.8)	100 (12.6)	148 (12.3)	
Moderate impairment	24 (7.8)	16 (4.4)	41 (5.2)	41 (3.4)	
Severe impairment	278 (9.0)	8 (2.2)	9 (1.1)	14 (1.2)	
Memory					
Short-term Free Recall					<0.0001
No impairment	160 (53.7)	259 (71.2)	551 (70.0)	956 (79.5)	
Mild impairment	39 (13.1)	38 (10.4)	97 (12.3)	113 (9.4)	
Moderate impairment	26 (8.7)	26 (7.1)	60 (7.6)	57 (4.7)	
Severe impairment	73 (24.5)	41 (11.3)	79 (10.0)	77 (6.4)	
Long-term Free Recall					<0.0001
No impairment	145 (48.8)	234 (64.3)	497 (63.2)	885 (73.6)	
Mild impairment	35 (11.8)	53 (14.6)	126 (16.0)	159 (13.2)	
Moderate impairment	33 (11.1)	35 (9.6)	87 (11.1)	74 (6.2)	
Severe impairment	84 (28.3)	42 (11.5)	76 (9.7)	85 (7.1)	
Executive Function					
Working Memory					<0.0001
No impairment	198 (65.8)	288 (78.7)	642 (81.1)	1005 (83.2)	
Mild impairment	52 (17.3)	54 (14.8)	100 (12.6)	148 (12.3)	
Moderate impairment	24 (8.0)	16 (4.4)	41 (5.2)	41 (3.4)	
Severe impairment	27 (9.0)	8 (2.2)	9 (1.1)	14 (1.2)	
Cognitive Initiation					
No impairment	156 (52.2)	252 (68.9)	513 (64.9)	890 (73.7)	
Mild impairment	62 (20.7)	74 (20.2)	157 (19.9)	219 (18.1)	
Moderate impairment	16 (5.4)	12 (3.3)	42 (5.3)	38 (3.2)	
Severe impairment	65 (21.7)	28 (7.7)	79 (10.0)	61 (5.1)	
Cognitive Flexibility					
No impairment	157 (53.4)	268 (73.6)	554 (70.8)	935 (77.7)	
Mild impairment	15 (5.1)	18 (5.0)	45 (5.8)	79 (6.6)	
Moderate impairment	19 (6.4)	18 (4.9)	46 (5.9)	50 (4.2)	
Severe impairment	105 (35.5)	60 (16.5)	138 (17.6)	139 (11.6)	
Visuospatial Organization					
Planning/Organization					<0.0001
No impairment	97 (36.7)	161 (51.1)	353 (49.9)	564 (53.0)	
Mild impairment	24 (9.1)	29 (9.2)	75 (10.6)	104 (9.8)	
Moderate impairment	20 (7.6)	19 (6.0)	64 (9.0)	95 (8.9)	
Severe impairment	123 (46.6)	106 (33.7)	216 (30.5)	302 (28.4)	

**Table 4:** Distribution of survivor health behaviors across patient activation levels.

Health Behavior	Level 1	Level 2	Level 3	Level 4	*p* Value
	No. (%)	No (%)	No (%)	No. (%)	
Meeting CDC Physical Activity Guidelines ^1^					<0.0001
Yes	129 (43.7)	182 (49.6)	465 (58.8)	813 (67.6)	
No	166 (56.3)	185 (50.4)	326 (41.2)	389 (32.4)	
Smoking ^2^					0.004
Never	212 (71.9)	253 (68.8)	554 (70.1)	915 (76.0)	
Current	53 (18.0)	64 (17.4)	142 (18.0)	147 (12.2)	
Former	30 (10.2)	51 (13.9)	94 (11.9)	142 (11.8)	
Risky drinking ^3^					0.0002
Yes	67 (23.2)	117 (32.3)	272 (35.0)	433 (36.6)	
No	222 (76.8)	245 (67.7)	505 (65.0)	749 (63.4)	
Sleep disturbance ^4^					0.42
None to slight	122 (40.5)	128 (34.8)	290 (36.6)	476 (39.4)	
Mild	151 (50.2)	198 (53.8)	432 (54.5)	614 (50.8)	
Moderate or Severe	28 (9.3)	42 (11.4)	70 (8.8)	118 (9.8)	
Healthy Eating Index score ^5^					<0.0001
<51 (poor diet)	93 (30.6)	102 (27.5)	198 (24.8)	213 (17.5)	
50–80 (needs improvement)	208 (68.4)	263 (70.9)	590 (73.8)	979 (80.2)	
>80 (good)	3 (1.0)	6 (1.6)	12 (1.5)	28 (2.3)	

^1^ CDC PA guidelines: endorsing 150 min of moderate or 75 min of vigorous PA per week. ^2^ Current smoking: within the past 30 days; former smoking: smoking outside of a 30-day period.^3^ Risky drinking: >3 per day or >7 per week (females) >4 per day or >14 per week (males). ^4^ PROMIS sleep disturbance 8a t-score <25 (none to slight), 25> and ≤30 (mild), and >30 (moderate to severe). ^5^ Healthy Eating Index score <51 (poor diet), 50–80 (needs improvement), and >80 (good).

**Table 5:** Distribution of survivor psychological factors across patient activation levels.

Psychological Factors	Level 1	Level 2	Level 3	Level 4	*p* Value
	No. (%)	No (%)	No (%)	No. (%)	
Depression symptoms ^1^					<0.0001
Yes	85 (28.2)	59 (16.0)	68 (8.5)	94 (7.7)	
No	216 (71.8)	310 (84.0)	728 (91.5)	1122 (92.3)	
Anxiety symptoms ^2^					<0.0001
Yes	71 (23.6)	43 (11.7)	67 (8.4)	83 (6.8)	
No	230 (76.4)	326 (88.3)	730 (91.6)	1132 (93.2)	
Somatization symptoms ^3^					<0.0001
Yes	88 (29.3)	56 (15.2)	83 (10.4)	103 (8.5)	
No	212 (70.7)	313 (84.8)	713 (89.6)	1112 (91.5)	
Cancer-Related Worry ^4^					
General fear					<0.0001
Yes	126 (41.9)	142 (38.5)	263 (33.0)	349 (28.7)	
No	175 (58.1)	227 (61.5)	534 (67.0)	868 (71.3)	
Body-focused					<0.0001
Yes	125 (41.5)	105 (28.4)	138 (17.3)	189 (15.5)	
No	176 (58.5)	265 (71.6)	659 (82.7)	1028 (84.5)	
Health-related quality of life ^5^					
Poor physical-health-related quality of life					<0.0001
Yes	113 (40.2)	79 (22.1)	119 (15.5)	114 (9.7)	
No	168 (59.8)	278 (77.9)	648 (84.5)	1058 (90.3)	
Poor mental-health-related quality of life					<0.0001
Yes	113 (40.4)	102 (28.7)	145 (18.9)	173 (14.8)	
No	167 (59.6)	253 (71.3)	622 (81.1)	999 (85.2)	

^1^ Brief Symptom Inventory (BSI) t-score ≥63 (top 10th percentile) (endorsing depression symptoms). ^2^ Brief Symptom Inventory (BSI) t-score ≥63 (top 10th percentile) (endorsing anxiety symptoms). ^3^ Brief Symptom Inventory (BSI) t-score ≥63 (top 10th percentile) (endorsing somatization symptoms). ^4^ Cancer-related worry (CRW) factor score <3 (not endorsing CRW) and ≥3 (endorsing CRW). ^5^ Short-Form Health Survey (SF-36) t-score score ≤ 40 (endorsing poor health-related quality of life).

**Table 6:** Multivariable logistic regression of physical activity level ^1^.

Independent Variables	Odds Ratio (OR)	95% CI
Patient Activation Level 2 vs. 1 3 vs. 1 4 vs. 1	1.14 1.53 2.07	0.80–1.62 1.13–2.09 1.53–2.80
Age at assessment	0.98	0.97–0.99
Age at diagnosis	0.99	0.97–1.01
Gender Male vs. Female	1.37	1.15–1.63
Diagnosis Group Lymphoma vs. Leukemia Sarcoma vs. Leukemia CNS Tumor vs. Leukemia Embryonal Tumor vs. Leukemia Other vs. Leukemia	0.90 0.75 0.69 0.79 0.86	0.68–1.18 0.56–1.00 0.51–0.92 0.58–1.06 0.61–1.22
Race NH Black vs. NH White Other vs. NH White	0.90 0.90	0.69–1.18 0.58–1.39
Educational Attainment Some post-high school vs. high school or less College graduate vs. high school or less	1.18 1.29	0.93–1.50 1.01–1.65
Intelligence Mild vs. no impairment Moderate vs. no impairment Severe vs. no impairment	0.91 0.61 0.59	0.64–1.28 0.38–1.00 0.37–0.95
Auditory condition grade 3+ at assessment Yes vs. No	0.97	0.74–1.26
Cardiovascular condition grade 3+ at assessment Yes vs. No	0.80	0.59–1.09
Endocrine condition grade 3+ at assessment Yes vs. No	0.78	0.65–0.93
Hematological condition grade 3+ at assessment Yes vs. No	0.32	0.06–1.69
Neurological condition grade 3+ at assessment Yes vs. No	0.52	0.35–0.76
Pulmonary condition grade 3+ at assessment Yes vs. No	0.66	0.47–0.93
Renal condition grade 3+ at assessment Yes vs. No	0.70	0.31–1.61

^1^ modeling probability of meeting CDC PA guidelines (150 min of moderate or 75 min of vigorous PA per week).

**Table 7:** Multivariable ordinal logistic regression of patient activation level ^1^.

Psychological Predictor	Number (%) of Total Sample	95% CI
Depression Symptoms ^2^	2682 (99.04%)	2.37 (1.87–2.99)
Anxiety Symptoms ^3^	2682 (99.04%)	2.21 (1.73–2.83)
Somatization Symptoms ^4^	2682 (99.04%)	1.99 (1.59–2.50)
Cancer-Related Worry		
General Fear ^5^	2684 (99.11%)	1.45 (1.23–1.71)
Body-Focused ^6^	2685 (99.15%)	2.21 (1.83–2.66)
Health-Related Quality of Life		
Physical Component ^7^	2577 (95.16%)	2.57 (2.06–3.20)
Mental Component ^8^	2574 (95.05%)	2.08 (1.72–2.52)

^1^ Modeling probability of endorsing higher ordered levels of patient activation; models adjusted for age at survey, age at diagnosis, race, educational attainment, gender, diagnosis, insurance status, perceived instrumental support, CTCAE grade 3+ auditory, cardiovascular, endocrine, immunologic, neurological, and pulmonary conditions. ^2^ Not endorsing depression symptoms (Brief Symptom Inventory (BSI) t-score ≥63 (top 10th percentile)). ^3^ Not endorsing anxiety symptoms (Brief Symptom Inventory (BSI) t-score ≥63 (top 10th percentile)). ^4^ Not endorsing somatization symptoms (Brief Symptom Inventory (BSI) t-score ≥63 (top 10th percentile)). ^5^ Not endorsing body-focused cancer-related worry (factor score ≥3). ^6^ Not endorsing body-focused cancer-related worry (factor score ≥3). ^7^ Not endorsing suboptimal physical health-related quality of life (Short-Form Health Survey (SF-36) t-score score ≤40)). ^8^ Not endorsing suboptimal mental health-related quality of life (Short-Form Health Survey (SF-36) t-score score ≤40).

## Data Availability

Data utilized for these analyses is available via Zenodo (doi: 10.5281/zenodo.13800123).

## Supplementary Materials

The following supporting information can be downloaded at: https://www.mdpi.com/article/10.3390/cancers16183220/s1, Table S1: Multivariable Logistic Regression of Smoking Behavior; Table S2: Multivariable Logistic Regression of Risky Drinking; Table S3: Multivariable Logistic Regression of Diet Quality; Table S4: Multivariable Logistic Regression of Sleep Disturbance1
