# Supplementary material for: Characterization of Patient Activation among Childhood Cancer Survivors in the St. Jude Lifetime Cohort Study (SJLIFE)
Source: Cancers (Basel). 2024 Sep 21;16(18):3220. doi: 10.3390/cancers16183220 (PMC11429751; doi:10.3390/cancers16183220)
Supplement: Supplementary file 1 [file cancers-16-03220-s001.zip › cancers-3175891-supplementary.pdf]

Supplementary Table S1. Multivariable Logistic Regression of Smoking Behavior[1]

Independent Variables	Odds Ratio (OR)	95% CI
Patient Activation Level		
2 vs. 1	1.11	0.68-1.80
3 vs. 1	1.10	0.72-1.68
4 vs. 1	0.87	0.57-1.32
Age at assessment	1.01	1.00-1.02
Age at diagnosis	0.98	0.95-1.00
Gender		
Male vs. Female	0.92	0.71-1.18
Diagnosis Group		
Lymphoma vs. Leukemia	1.09	0.74-1.62
Sarcoma vs. Leukemia	1.34	0.89-2.00
CNS Tumor vs. Leukemia	0.66	0.42-1.05
Embryonal Tumor vs. Leukemia	1.42	0.97-2.10
Other vs. Leukemia	1.31	0.80-2.13
Race		
NH Black vs. NH White	0.67	0.45-0.99
Other vs. NH White	0.51	0.24-1.05
Educational Attainment		
Some post-high school vs. High school or less	0.67	0.50-0.91
College graduate vs. High school or less	0.27	0.19-0.39
Intelligence		
Mild vs. No impairment	1.12	0.72-1.75
Moderate vs. No impairment	1.00	0.52-1.90
Severe vs. No impairment	1.04	0.56-1.91
Perceived Instrumental Support	0.98	0.97-0.99
Endocrine Condition Grade 3+ at assessment		
Yes vs. No	0.75	0.57-0.97
Gastrointestinal Condition Grade 3+ at assessment		
Yes vs. No	1.51	0.61-3.72
Immunologic Condition Grade 3+ at assessment		
Yes vs. No	1.94	0.71-5.31
Neurological Condition Grade 3+ at assessment		
Yes vs. No	0.75	0.41-1.40
Ocular Condition Grade 3+ at assessment		
Yes vs. No	0.70	0.44-1.09

[1]modeling probability of endorsing smoking (current use within past 30 days)

Supplementary Table S2. Multivariable Logistic Regression of Risky Drinking[1]

Independent Variables	Odds Ratio (OR)	95% CI
Patient Activation Level		
2 vs. 1	1.38	0.92-2.09
3 vs. 1	1.50	1.04-2.15
4 vs. 1	1.41	0.99-2.02
Age at assessment	0.98	0.97-0.99
Age at diagnosis	1.02	1.00-1.04
Gender		
Male vs. Female	1.43	1.19-1.73
Diagnosis Group		
Lymphoma vs. Leukemia	1.22	0.92-1.62
Sarcoma vs. Leukemia	1.36	1.00-1.85
CNS Tumor vs. Leukemia	0.63	0.46-0.87
Embryonal Tumor vs. Leukemia	1.35	0.99-1.85
Other vs. Leukemia	0.80	0.54-1.16
Race		
NH Black vs. NH White	0.43	0.31-0.60
Other vs. NH White	0.66	0.41-1.06
Educational Attainment		
Some post-high school vs. High school or less	1.51	1.15-2.25
College graduate vs. High school or less	1.71	1.30-1.73
Intelligence		
Mild vs. No impairment	0.69	0.46-1.03
Moderate vs. No impairment	0.38	0.18-0.78
Severe vs. No impairment	0.66	0.34-1.27
Perceived Instrumental Support	0.99	0.98-0.99
Auditory Condition Grade 3+ at assessment		
Yes vs. No	0.78	0.58-1.06
Endocrine Condition Grade 3+ at assessment		
Yes vs. No	0.78	0.64-0.95
Gastrointestinal Condition Grade 3+ at assessment		
Yes vs. No	0.60	0.24-1.53
Immunologic Condition Grade 3+ at assessment		
Yes vs. No	0.31	0.09-1.09
Neurological Condition Grade 3+ at assessment		
Yes vs. No	0.71	0.44-1.15
Ocular Condition Grade 3+ at assessment		
Yes vs. No	0.63	0.45-0.89
Pulmonary Condition Grade 3+ at assessment		
Yes vs. No	0.94	0.65-1.36

Renal Condition Grade 3+ at assessment		
Yes vs. No	0.12	0.02-0.92

[1]modeling probability of endorsing risky drinking (>3 per day or >7 per week (females) >4 per day or >14 per week (males))

Supplementary Table S3. Multivariable Logistic Regression of Diet Quality[1]

Independent Variables	Odds Ratio (OR)	95% CI
Patient Activation Level		
2 vs. 1	1.05	0.24-4.51
3 vs. 1	1.19	0.33-4.32
4 vs. 1	1.53	0.45-5.25
Age at assessment	1.02	0.98-1.05
Age at diagnosis	0.99	0.93-1.05
Gender		
Male vs. Female	0.64	0.35-1.18
Diagnosis Group		
Lymphoma vs. Leukemia	0.78	0.33-1.88
Sarcoma vs. Leukemia	0.46	0.15-1.41
CNS Tumor vs. Leukemia	0.55	0.18-1.71
Embryonal Tumor vs. Leukemia	0.34	0.10-1.18
Other vs. Leukemia	1.28	0.50-3.31
Race		
NH Black vs. NH White	0.72	0.24-2.10
Other vs. NH White	1.95	0.65-5.88
Educational Attainment		
Some post-high school vs. High school or less	0.84	0.31-2.29
College graduate vs. High school or less	2.39	1.03-5.54
Insurance Status		
Insured vs. Uninsured	0.38	0.17-0.84
Endocrine Condition Grade 3+ at assessment		
Yes vs. No	0.46	0.22-0.94

[1]modeling probability of endorsing good diet quality (Healthy eating index score >80)

Supplementary Table S4. Multivariable Logistic Regression of Sleep Disturbance[1]

**Independent Variables**	**Odds Ratio (OR)**	**95% CI**
Patient Activation Level		
2 vs. 1	1.13	0.80-1.61
3 vs. 1	1.07	0.79-1.46
4 vs. 1	0.87	0.65-1.17
Age at assessment	1.00	0.99-1.01
Age at diagnosis	1.00	0.98-1.01
Gender		
Male vs. Female	0.99	0.83-1.18
Diagnosis Group		
Lymphoma vs. Leukemia	1.02	0.79-1.33
Sarcoma vs. Leukemia	1.24	0.93-1.66
CNS Tumor vs. Leukemia	0.87	0.66-1.15
Embryonal Tumor vs. Leukemia	1.05	0.78-1.41
Other vs. Leukemia	0.92	0.67-1.28
Race		
NH Black vs. NH White	0.99	0.76-1.28
Other vs. NH White	0.82	0.54-1.25
Educational Attainment		
Some post-high school vs. High school or less	1.15	0.91-1.45
College graduate vs. High school or less	1.38	1.09-1.76
Intelligence		
Mild vs. No impairment	1.14	0.81-1.60
Moderate vs. No impairment	1.12	0.68-1.83
Severe vs. No impairment	0.73	0.46-1.14
Gastrointestinal Condition Grade 3+ at assessment		
Yes vs. No	2.38	1.02-5.56

[1]modeling probability of endorsing none to slight sleep disturbance (PROMIS Sleep disturbance t-score <25)
